# The hermeneutics of symptoms

**DOI:** 10.1007/s11019-022-10086-z

**Published:** 2022-05-03

**Authors:** Alistair Wardrope, Markus Reuber

**Affiliations:** 1 Department of Neuroscience, The University of Sheffield, Sheffield, UK; 2 Department of Clinical Neurology, Sheffield Teaching Hospitals NHS Foundation Trust, Sheffield, UK

**Keywords:** Phenomenology, Symptoms, Hermeneutics, Narrative medicine, Subjectivity

## Abstract

The clinical encounter begins with presentation of an illness experience; but throughout that encounter, something else is constructed from it – a symptom. The symptom is a particular interpretation of that experience, useful for certain purposes in particular contexts. The hermeneutics of medicine – the study of the interpretation of human experience in medical terms – has largely taken the process of symptom-construction to be transparent, focussing instead on how constellations of symptoms are interpreted as representative of particular conditions. This paper examines the hermeneutical activity of symptom-construction more closely. I propose a fourfold account of the *clinical* function of symptoms: as theoretical entities; as tools for communication; as guides to palliative intervention; and as candidates for medical explanation or intervention. I also highlight roles they might play in illness experience. I use this framework to discuss four potential failures of symptom-interpretation: failure of symptom-type and symptom-token recognition; loss of the complete picture of illness experience through overwhelming emphasis on its symptomatic interpretation; and intersubjective feedback effects of symptom description altering the ill person’s own perceptions of their phenomenal experience. I conclude with some suggestions of potential remedies for failures in the process of symptom-construction.

## Introduction

A person has an unpleasant experience. They interpret this experience in bodily terms. They seek medical attention, encounter a clinician. The clinician asks: what is wrong with you? They respond: a nagging, burning discomfort, felt most intensely behind the breast bone, but spreading into the back of the throat. The clinician makes note of this: ‘Presenting complaint: chest pain’.

This translation is so commonplace in medical practice as to be almost transparent: the clinician presenting such a case to their colleague will rarely prompt a response, ‘how do you know they have chest pain?’ Yet in moving from the ‘nagging, burning discomfort’ to ‘chest pain’, the clinician has collaborated in turning the account of human experience into something else – a *symptom*. This transformation is not merely paraphrase – the symptom of chest pain has a range of powers that the discomfort does not. To a triage call handler, it can prompt the calling of an ambulance. To a ward nurse, it can necessitate urgent doctor review. For that doctor, it might demand an ECG before they even begin the review.

The mundanity of this interpretive activity belies its complexity. Before even arriving at the clinical encounter, the ill person has undergone an iterative process of interpretation, articulation, and social negotiation to decide whether their sensation merits medical attention (Hay 2008; Heritage 2009; Heritage and Robinson 2006). The experience is presented in the clinical encounter, but that presentation is guided not only by the experience itself and the patient’s articulation of it, but the clinician’s “channelling” of the discourse (Leder 1990). The directions of this channelling are shaped by the clinician’s medical knowledge, clinical experience, and their perception of the purpose of the encounter – which perception may differ from that of the patient (Svenaeus 2000). Not only does this co-construction shape the remainder of the clinical encounter; it provides another interpretive iteration through which the patient presents their experience (when they see another clinician, they might open with: I have a chest pain here^1^) (Heritage and Robinson 2006).

It is unsurprising, then, that such a complex activity might regularly go wrong. The recent reignition of interest in the phenomenology of illness, and in the failures of testimonial exchange in the clinical encounter, raise a number of concerns relating to this basic interpretive activity of clinical medicine. Experiences may not be translated effectively, with a failure to identify clinically relevant symptoms (Carel and Kidd 2014; Fricker 2009; Saks 2008), or to infer their presence inappropriately (Crichton et al. 2017). Whole classes of experience – though shared between multiple ill persons, and potentially amenable to biological explanation – may not be recognised as symptoms at all (Fricker 2009; Lakeman 2010). The framework of ‘symptoms’ itself may be inadequate to capture some aspects of the experience, being predicated on modelling the body as an object – neglecting the inherently first-personal ‘lived’ body (Buchman et al. 2017; Carel 2016; Carel and Kidd 2014; Freeman 2015). Or – as the quote from Sartre above suggests – the very act of translation may warp that being translated, giving us no means of interpreting our experiences other than as symptoms (Foucault 2003; Illich 1982; Wardrope 2015).

Addressing these concerns will require understanding: how symptoms are produced; why they are produced (what functions they serve); the mechanisms that might disrupt this production; and the goals of the clinical encounter. I will argue that, *contra* other works in the ‘hermeneutics of medicine’ (the project of engaging with medical practice as an interpretive activity), the basic interpretive activity of the clinical encounter is that of turning sensation into symptom. I do so by first introducing some basic theoretical tools of hermeneutics (§ 2.1) and their medical application (§ 2.2), using these to show that the translation of sensation into symptom is an interpretive act that existing approaches to the hermeneutics of medicine take for granted (§ 2.3). After establishing the prerequisites for an experience to be brought to the clinical encounter as a candidate symptom (§ 3), I describe symptom construction through examining its functions (§ 4). I propose a fourfold *clinical* role for this activity (§ 4.1):^2^ enabling communication between health workers; enabling systematisation within medical models of bodily phenomena; making experiences a candidate for such systematisation; or guiding alleviation of experience through palliative interventions. These functions are guided by the ends of the medical encounter itself. However, symptomatisation may play different roles for the *ill person* (§ 4.2). This framework allows us to understand how the interpretation of symptoms may go wrong – or, even when conducted appropriately, create a source of tension in the clinical encounter (§ 5). Addressing the purported failures in the interpretive activity of symptom construction will require training clinicians to acknowledge their epistemic limitations and to have the curiosity and humility to go beyond them; equipping clinicians and ill persons with the tools to support expression of their experiences; and accepting that medicine itself cannot and should not attempt to describe or understand all of illness experience without remainder (§ 6).

## The hermeneutics of medicine and the hermeneutics of symptoms

### Hermeneutics in medicine

In the opening example, we portray a clinician constructing a symptom – that of chest pain – out of a patient experience – the ‘nagging discomfort’. We described this as an interpretive activity. This, broadly speaking, puts the subject of this paper into the field of the ‘hermeneutics of medicine’ – the project of understanding medical practice in terms of acts of interpretation.

The label of ‘hermeneutics’ is multivocal, and – depending on context and interests – could describe a variety of approaches or traditions (Bernstein 1983; Daniel 1986). The most influential applications of hermeneutics in medicine have drawn on the ideas of hermeneutical phenomenology, as embodied in the works *inter alia* of Hans-Georg Gadamer and Paul Ricoeur (perhaps unsurprisingly; both were students of the physician Karl Jaspers). While it is beyond the scope of this article to address this tradition in detail, I suggest some principles of interpretation relevant to medical practice.

#### Interpretation as a matter of perspective

In medieval biblical hermeneutics, understanding could be broken down into distinct activities: identifying the literal facts; determining what to believe on the basis of those facts; and settling on their application (Bernstein 1983). Drawing on Aristotle’s concept of practical wisdom (*phronesis*), Gadamer argues instead for an approach that considers interpretation and application inseparable (Gadamer 2013). The range of interpretations of any given phenomenon (whether that be an experience, an utterance, or a piece of text) are potentially inexhaustible. Different aspects of that phenomenon may also be more or less salient to different agents. The question of the ‘best’ interpretation is not one that can be settled by procedural or methodological considerations alone; it will depend upon the uses to which that understanding is being put (Bernstein 1983; Svenaeus 2003).

Fredrik Svenaeus, following Gadamer, calls the hermeneutics of medicine ‘applicative’ (Svenaeus 2000, p. 180) – the interpretations offered are “put to work in a certain setting with a specific goal.” This has the implication that understanding the hermeneutics of medical practice will depend heavily on the ends to which those interpretations are used.

#### Prefiguration and prejudice

A second key principle of phenomenological hermeneutics is that our historical and social context conditions our interpretations. Our perceptions of the world come not in the form of undifferentiated, raw sense data, but of things in the world – “all consciousness is consciousness of something” (Merleau-Ponty 2002, p. 6). As Heidegger puts it: “We never really first perceive a throng of sensations, e.g. tones and noises … rather we hear the storm whistling in the chimney, we hear the three motored plane … we hear the door shut in the house” (Gallagher and Zahavi 2020, pp. 126–7). The prior conceptions that colour our perceptions and interpretations are conditioned by the conceptual tools available to us in our particular contexts, in what Gadamer refers to as ‘prejudice’, and Ricoeur ‘prefiguration’. To say that our interpretations are ‘prejudiced’ in this fashion is not to state they are erroneous (Bernstein 1982, p. 826); it is simply an honest acknowledgement of how we experience the world. As Mikhhail Bakhtin puts it, “only the mythical Adam, who approached a virginal and as yet verbally unqualified world” (Bakhtin 1981, p. 279) could escape such conditions.

The hermeneutical emphasis on prior conditions of understanding is increasingly supported by the neuroscience of perception. As demonstrated in predictive coding accounts of perceptual processes, these are the result of a constructive process, in which sensory information is interpreted through the lens of predictions, with our experience comprising a model of the world that is constantly revised with a view to minimising predictive error (Edwards et al. 2012; Van den Bergh et al. 2017). Acknowledging the role of prefiguration in medical interpretation draws attention to how existing conceptual resources may shape our understanding and communication of bodily experience – in ways that may illuminate or obscure certain aspects of that experience.

#### Incompleteness of interpretation

While prior conditions shape interpretation, however; they do not determine it completely. Perceptions may still surprise us – to return to Heidegger, while we may usually hear the door shut in the house, sometimes we start at a loud bang and think only ‘what was that’? Moreover, an answer to that question need not tell the whole story: the answers ‘rapid combustion-induced gas expansion’, ‘a mistimed ignition spark’, and ‘a car backfiring’ might all be appropriate, depending on one’s perspective. This idea should be familiar to anyone acquainted with medical practice, in which non-overlapping (and sometimes contradictory) descriptions of phenomena can be employed in different contexts (contrast the picture of blood constituents and flow used to model hypertension, peripheral oedema, or venous thromboembolism) (Wardrope 2017).

Ricoeur refers to the incompleteness of any given interpretation as the “surplus of meaning” (Ricoeur 1976); it provides a counterbalance to the unifying, systematising effect of our prefigurations. In Bakhtin’s metaphor, it provides a ‘centrifugal force’ to balance the ‘centripetal forces’ of the order our prior conceptual frameworks impose on the world (Bakhtin 1981, p. 272). While the centripetal forces of our preferred conceptual resources encourage interpretation of phenomena in certain terms, to an extent this will always involve trying to fit square pegs into round holes.

#### The hermeneutical circle

This last observation – that no interpretation of the world is able to carve nature at the joints, without remainder – leads to the last, and perhaps most widely-known, relevant idea drawn from philosophical hermeneutics – that of the hermeneutical circle (Gadamer 2013, p. 279). The tension between a given interpretation and the surplus of meaning – between centrifugal and centripetal forces – makes interpretation an iterative process: our interpretations are shaped by our prior expectations, and those expectations are then revised in light of new information. Furthermore, this revision is intimately bound with our responses to our interpretations – the use we make of that information shapes what is most salient in our updated expectations (Bakhtin 1981, p. 282; Barker 2017; Bernstein 1982).

The hermeneutical circle can be understood in neuroscientific terms using a predictive coding model. Our prior expectations – our existing model of the world – shape our perceptions, but does not account for them without remainder; our model is then updated to minimise this prediction error (Edwards et al. 2012; Van den Bergh et al. 2017). Furthermore, such updating is intimately bound to our responses to this information, insofar as we can minimise prediction error through ‘active inference’ – either changing predictions to explain sensory input, or (through action) changing sensory input to match those predictions (Brown et al. 2013).

The hermeneutical circle brings together the first three features in providing a model of the interpretive process: our interests and prior expectations shape – but do not determine – our interpretations, and the consequent interpretations (both in what they cover and what they leave out) in turn re-shape our expectations and interests.

### Hermeneutics of medicine

Various authors have sought to apply these tools to understanding medical practice. In one early account, Daniel models medical practice as analogous to reading a literary text: a patient relates their symptoms, and shows bodily signs; the clinician interprets these as pointing to a given diagnosis; patient and clinician act on the basis of this interpretation, effecting change in their corresponding worlds (Daniel 1986). A very different hermeneutical approach can be found in psychoanalytic and psychodynamic models of pathology, which interpret symptoms as expressing drives or achieving ends of which the sufferer is unconscious (Snyder and Smith 1982). Others have found these unsatisfactory – the former as too caught up in the centripetal force of medical discourse, the ‘hermeneutics of suspicion’ embodied in the latter as failing to acknowledge the surplus of meaning in its parsing of symptoms as *just* the expression of unconscious wishes (Shirley 1977; Svenaeus 2000). More nuanced accounts describe the clinical encounter as a dialogue between doctor and patient, directed at understanding the patient’s experience of illness, how it (re)shapes their being in the world, and how medical technologies may be used to intervene in it (Leder 1990; Svenaeus 2000).

The specifics of these accounts lie beyond the scope of this paper. For my purposes, it is relevant to note simply that all of the above take for granted the act of interpretation with which this paper opens. Symptoms are *things to be interpreted*, not *results of interpretation*; they are taken to be transparently accessible to the ill person, even if they take some effort in articulation. The transition from ‘nagging, burning discomfort’ to ‘chest pain’ goes as unnoticed in these accounts as in the average consultation.

For example, consider Frederick Svenaeus’ narrative of ‘Jane’, a 55-year old woman who, through the course of his book, comes to be given a diagnosis of type 2 diabetes mellitus. From the start, Jane is able to describe that she “would get terribly thirsty”, had urinary urgency, “blurred vision” and “lost weight”(Svenaeus 2000, p. 96). Her doctor friend is able to recognise the *symptoms* immediately, without comment – the interpretive activity concerns how these relate to her subsequent diagnosis, and the effect of that diagnosis on her being-in-the-world. Similarly, in Drew Leder’s account, the ‘experiential text’ (the “series of experiences which stands out as significant and disruptive”(Leder 1990, p. 11)) features experiences already intelligible as symptoms, the interpretation coming in constructing a ‘plot’ from these ‘scraps of pages’: “When I go to the doctor for *abdominal* problems, the *intermittent cramps* and *acid reflux* are like a page, a scrap of a page, from which I seek to reconstruct a comprehensive plot.” (Leder 1990, pp. 12–13) (My emphasis).

### Hermeneutics of symptoms

I wish to focus on another, more basic form of medical hermeneutics – the interpretive acts by which ‘symptoms’ are created - the change from “felt experience” into a “constructed and socially informed cognitive interpretation”(Eriksen and Risør 2014). By suggesting that ‘symptoms’ are constructed in the clinical encounter, I do not wish to propose that the patient is not experiencing anything before that encounter; there is clearly some illness experience that (in the majority of cases) motivates the encounter taking place at all. Rather, it is the translation of that experience to a certain kind of conceptual entity – one that has a home in medical discourse and models of human phenomena – that takes place in the encounter.

To make this distinction clearer, contrast Leder’s abdominal problems quoted above with a very different phenomenological narration of what, ultimately, might be considered similar symptoms. In accounting for the construction of dyspepsia from a stomach ulcer, Sartre observes that – in the immediate, first-person experience – the stomach itself does not even feature:

*[B]efore the intervention of the alienating, cognitive stratum, the pain is neither a local sign nor identification … At this level, ‘the stomach’ is an inexpressible; it can neither be named or thought, it is only this suffered figure which is raised on the ground of the body-existed.* (Sartre 1978, p. 355)

The representation of the pain being ‘in the stomach’ requires refracting the experience through a particular lens: the “objectivating empirical knowledge” of the clinician (or at least a lay biological model whose conceptual resources are shaped heavily by the clinician’s). Constituting the illness in such terms in fact constitutes “directions of flight” from which the illness experience “escapes me.” In this process, the experience is transformed:

*At this point a new layer of existence appears: we have surpassed the lived pain toward the suffered illness; now we surpass the illness toward the Disease … Others have informed me of it, Others can diagnose it; it is present for Others … Its true nature is therefore a pure and simple being-for-others.* (Sartre 1978, p. 356)

In Sartre’s terminology, the symptom is constructed by the movement from the subjective ‘body-for-me’, into the objective, third-person ‘body-for-the-Other’.^3^ This movement is an interpretive one: the medical prefiguration shapes the ‘inexpressible’ pain in terms relating to bodily organs; those terms provide a centripetal organising force for our understanding, but in so doing create “a new layer of existence”, that can be used by others, for certain purposes (e.g. diagnosis). When Leder writes of ‘acid reflux’ as a scrap in the pages of the ‘experiential text’, he is bypassing the stage of interpretation required to turn experience itself into that scrap – to make a poorly-localised burning, an unpleasant taste on lying down, a post-prandial feeling of unrest, into ‘reflux’. It is this interpretive activity – the hermeneutics of symptoms, rather than medicine – on which I wish to focus.

## From sensation to symptom

In the Introduction, we discussed the case of the ill person presenting to a clinician with a ‘nagging, burning discomfort’; my focus will be its transformation into the symptom of ‘chest pain’ – but before even reaching this point, an interpretive effort has taken place for it to be considered a candidate for medical explanation or intervention.

Firstly, the very fact of perceptual awareness is not straightforward. Such awareness is not dichotomous, but can permit gradations (Jimenez et al. 2020). Beyond this, identifying the precise characteristics of one’s subjective experience is no trivial activity: we regularly fail to devote attention to the experience, or else the act of paying attention to it produces representational distortions of the experience itself (Petitmengin 2006); or associated emotional appraisals may influence attentiveness to particular aspects of the experience (Van den Bergh et al. 2017). As already referenced, perception is a constructive process that depends as much on our expectations – or prefigurations, to follow Ricoeur – as sensory inputs (Edwards et al. 2012; Van den Bergh et al. 2017).

Beyond awareness and articulation of the experience, the ill person then has to conceive of themselves as such – to think that the experience represents illness. The process by which this occurs has been explored from several perspectives, but all converge on ideas of disruption, loss of continuity and the taken-for granted. Based on ethnographic research within a community on the Indonesian island of Lombok, Cameron Hay presents a model whereby the sense of *disability* – disruption of the ability to perform one’s normal daily activities – and *vulnerability* –awareness of one’s fragility and potential for harm – are intimately tied to their becoming potential symptoms (Hay 2008). Interviews with people with lung cancer in Denmark, England and Sweden support this model (Bernhardson et al. 2021). Phenomenology of illness refines these accounts: whether as loss of wholeness, certainty, control, freedom, and the familiar (Toombs 1992); or the intrusion of ‘bodily doubt’ – estrangement and detachment from a pre-reflective sense of embodied agency (Carel 2016). I cannot do justice to these accounts here: for our purposes, the point is that experiences do not straightforwardly and readily announce themselves as those of ‘illness’.

The presentation of such experiences in the clinical encounter is furthermore mediated by social evaluation and negotiation. Hay’s work demonstrates his informants presenting their experiences to family and friends, seeking intersubjective validation and testing their legitimacy as illness experience, before accessing more formal health care (Hay 2008). Mette Bech Risør identifies a similar process at play in a series of interviews with participants with functional disorders in Norway (Risør 2011). Different communities within societies operate on different standards of ‘candidacy’ for explanation of experience in terms of illness, affecting rates of health care access (Dixon-Woods et al. 2006). Linguistic analysis of initial healthcare consultations demonstrate the work done by patients to represent their problems as ‘doctorable’ – appropriate material for consideration in a healthcare context (Heritage 2009; Heritage and Robinson 2006).

Only having undergone this process can the ill person then begin to present their experience in the clinical encounter, and thus potentially participate in the construction of a symptom from their experience. As discussed above, such interpretation presupposes a purpose. To understand this further, then, we will have to explore the perspectives and purposes of the participants in symptom construction: to interrogate the functions of symptoms.

## The functions of symptoms

Risør, in her study of the process of symptom-making in people with functional disorders, describes symptoms as “commodity-actants” – they have a specific social value that can be used for particular ends in certain contexts (a commodity); and they act to change the social context in which they arise, demanding specific responses (an actant). For example, return again to our ‘niggling discomfort’ - when interpreted as the symptom ‘chest pain’, it acts on those interpreting it; a triage handler or nurse may have to ensure urgent medical review is arranged, an ill person in the Emergency Department may need more prompt assessment. And it has an exchange value – an assessing clinician can readily acquire tests (e.g. an ECG) that in other contexts might not be immediately available. These actions, and this value, would not similarly be available if the discomfort were instead labelled ‘reflux’ or ‘indigestion’.

Moreover, the roles played by symptoms may differ in different contexts and from different perspectives. Symptoms, in the sense in which I am using the term, are how medical discourse translates human experience. I therefore focus first on the uses clinicians make of these interpretations. However – while (as explored further below) they certainly do not say all that is relevant about illness experience, ill people nonetheless may make use of them – sometimes in directions that are tangential, or even opposed to, clinicians’ use. One could further consider their function from a third perspective – that of their social role (analogous to third component of the tripartite model of disease, illness, and sickness (Hofmann 2002)) but I shall focus here on the parties to the clinical encounter directly engaged in the act of symptom interpretation.

### Clinical functions

From the clinician’s perspective, symptoms can be considered tools to further the purpose of the clinical encounter. This is somewhat complicated by the lack of clear agreement on what that purpose is. While there is frequent assertion that the objective is of achieving a healing action (Pellegrino 2001), or of restoring health (Svenaeus 2000), these proposals are troubled by more than just the devil in the details of health’s definition: they are also simultaneously too broad, and too narrow. Too broad, because many other forms of human interaction could be thought to be directed at achieving healing – from personal trainer to conflict mediator. Too narrow, because not all clinical encounters need be directed at health: consider the terminal diagnosis.

An alternative approach is to consider the social position of health workers and institutions, and derive a purpose thence. In this light, health workers are members of a profession; they provide certain forms of expertise in return for a degree of social licence (Wynia 2008). In common with other experts, the function of our encounters with them is to utilise their expertise toward resolution of certain human problems (Heritage 2009). In what, then, does (contemporary, industrialised, ‘Western’) clinician expertise lie? At a first pass, we may say something like this: clinician expertise lies in understanding human bodily phenomena in objective terms: in human anatomy, physiology, psychology and the other disciplines of the biomedical sciences; in interpreting people’s experience in these terms (i.e. diagnosis); and using these models to make predictions about people’s future trajectories (prognosis), and intervening to change those trajectories (management).

No doubt this definition will prove to be incomplete, but as a sketch it has some important features. It avoids the traps of narrowness and broadness of pure healing-based definitions, by both restricting the scope of the kinds of healing that could be considered medical, and acknowledging that clinical expertise need not always be directed toward healing alone. In emphasising the role of the biomedical sciences in medicine’s own self-conception of its expertise, it highlights a feature already referenced above (in moving from Sartre’s inexpressible gastralgia through “objectivating clinical knowledge” to stomach ache) and that will become important again later – the emphasis on *body as object*. Lastly, it makes clear that clinical expertise does lie in understanding human phenomena in certain terms – that it requires an act of interpretation. How, then, can symptoms be used to serve this purpose?

#### Symptoms as theoretical entities

The first, and most obvious, example, is that of rendering the subjective experience into terms intelligible in pathophysiological models. Any scientific model will be described in terms of certain entities, and activities in which they can participate, laws that govern their behaviour, or patterns of development of the systems comprising those entities. But to apply such models requires identification of its components with phenomena in the domain of concern (Giere 2010; Suárez 2010). Just as ‘the source of the curved trail in the cloud chamber’, ‘that which is scattered off deuterium to investigate the weak neutral current’, and ‘that which, when fired one-by-one at a double slit, cumulatively produce a wave-like interference pattern’ are all represented by the theoretical entity ‘electron’, so too must a range of different descriptions of experience be identified with particular symptoms.

Symptoms can be present within medical models in a range of fashions. Classically, they are the *explananda* – as when the ‘niggling discomfort’ is interpreted as ‘reflux’ so that it may be explained by stomach acid flowing proximally into the oesophagus, the resulting irritation stimulating somatic afferent nerve firing that produces the ‘reflux’ sensation. But they may also constitute (at least in part) disease entities themselves, as with contemporary ‘network’ approaches to mental disorder (Borsboom 2017) (and, arguably, long-standing ‘syndromic’ diagnoses).

#### Symptoms as guide to palliative intervention

The clinician need not, however, always see the body-object through the lens of a fleshed-out medical model in order to intervene. Clinicians and patients may use ‘black-box’ symptomatic control strategies for complaints as diverse as pain, nausea, vertigo, insomnia, or tremor. However, a sensation needs to be identified with one of these categories before such strategies may be employed.^4^

Of course, all these symptoms may also be approached by trying to explain them in light of biomedical knowledge, and targeting treatments mechanistically – the point here is simply that one need not do so. One can name an experience as ‘symptom’, test whether a treatment ameliorates that symptom – even while saying *hypotheses non fingo* as regards *why* it might do so – and, if successful, apply it to similar future experiences identified with the same symptom.^5^

#### Symptom as tool for communication

Much of the extant literature on the nature of clinical practice and the ends of medicine takes its paradigmatic event to be the dyadic encounter between clinician and patient. However – at least in contemporary industrialised healthcare settings – this is the exception rather than the rule. The construction of the medical image of the ill person is iterative and multidisciplinary.

Consider the following trajectory of an acutely ill person: Mary is having her breakfast when she notices that *she can’t pick up her teacup*. She calls for her partner’s help, but *her words come out funny*. Her partner calls an ambulance, and paramedics shortly arrive. From Mary and her partner they determine that Mary has developed *lateralising weakness* and *speech disturbance*, and from their brief examination find she is *F.A.S.T.* (Face, Arms, Speech, Time [to call 999]) *positive*. The paramedics speak to a Stroke Unit and she is accepted for immediate admission, where the assessing clinician confirms *right-sided face, arm, and leg weakness* and *expressive dysphasia*. She describes this to the radiologist, who vets and reviews a CT of Mary’s head looking for a suspected stroke, identifying a vessel hyperdensity in keeping with a left middle cerebral artery occlusion.

In this account, the translation of Mary’s experience (‘can’t pick up teacup’ and ‘words come out funny’) into symptoms (‘right-sided weakness’ and ‘expressive dysphasia’) makes them suitable for incorporation into a medical model (consequences of a dominant-hemisphere ischaemic stroke). But before that point, the symptomatic translations play a crucial communicative role amongst the multi-disciplinary team involved in her initial assessment and management. By parsing her experience in such terms (summarised as being ‘FAST positive’) the paramedics present a case that the Stroke Unit must accept her immediate admission. The clinician, in describing the pattern of weakness and dysphasia to the radiologist, both justifies the need for brain imaging and suggests where they should be looking for abnormalities. In these exchanges, the symptoms act as commodities. In high-intensity, high-pressure healthcare settings, there is always incentive to manage workload by rejecting admissions as unnecessary or investigations as not clinically indicated. But these specific symptoms are effective bargaining chips in negotiating for the course of action the assessor feels indicated. This reflects their role as actants – the context of evidence, guidelines, and institutional norms within which health care workers operate mean that certain symptoms require certain courses of action (absent very good reason to believe e.g. the investigation would be unsafe or futile, if the clinician had parsed Mary’s symptoms as in the anecdote above and yet not ordered the CT she would be considered negligent).^6^

Contrast Mary’s case with that of some people with functional symptoms interviewed by Mette Bech Risør. Their experiences could not readily be translated into symptoms with a particular exchange value in bargaining for referrals, investigations, or treatments; their “sensations … somehow do not fit into the doctors’ toolkit” (Risør 2011); as a consequence, the symptomatisation of their experience – and further medical management – is called to a halt.

#### Symptom as candidate for medical intervention or explanation

The above functions work on the assumption of a symptom already recognised and incorporated into medical models and discourse. But every symptom has to enter that discourse somewhere; particular ways of being must be identified as potential candidates for fulfilling one of the above functions.

The construction of ‘epileptic seizures’ demonstrates this process in action. Epileptic seizures are transient occurrences of behavioural and/or experiential alterations produced by excessive and hypersynchronous neuronal activity in the brain. Epilepsy is a condition in which people are predisposed to experience unprovoked seizures. It is the nature of the condition that these seizures are recurrent, and that they are highly stereotyped. Thus people with epilepsy will repeatedly encounter a particular set of experiences over the course of months or years. When seizures originate in networks within one region of the brain – confined to one ‘hemisphere’ – they are known as focal seizures. When they emerge more or less symmetrically and simultaneously in bilateral networks throughout the brain, they are known as generalised. Both types of epileptic seizures may involve loss of consciousness, which may be the initial seizure symptom. However, especially focal epileptic seizures may also involve retained consciousness and be compatible with a range of levels and contents of awareness. Seizure symptoms can evolve during individual seizures and awareness may be lost as the ictal neuronal activity in the brain spreads from regionally confined networks to more widely disseminated networks. Ultimately, both focal and primary generalised seizures can develop into so-called “bilateral tonic clonic seizures” which invariably involve loss of awareness and self-control. In such situations, the symptoms of the initial phase of the seizure – before the more dramatic bilateral stage and onset of impairment of awareness – are sometimes known as an ‘aura’.

The etymology of this term demonstrates the process of experiences becoming treated as candidates for medical explanation or intervention in action. The writings of the Greek physician Galen document his encounter with a thirteen-year old boy who experienced convulsions. On being asked about his condition, the boy described a sensation starting in his leg, climbing upward towards the head, at which point a bilateral convulsion would result. The entity supposedly doing the climbing he described as a ‘breeze’, or *αὔρα* (Temkin 1994, p. 37).

It is through identifying experiences such as these that Galen’s writings create a model of that which was to be explained by medicine. The idea of aura – “not, originally, a scholarly or theoretical idea but one that was brought into medicine by way of the patients’ complaints”(Lardreau 2007) – permitted the construction of a pathophysiological model of epileptic seizures as resulting from a rising substance that, when it reached the head, would result in loss of consciousness. This in turn guided mooted treatments, such as applying ligatures to limbs affected by focal seizures to arrest the spread of this substance (Temkin 1994, p. 38).

In this case we see that, although not yet incorporated into the conceptual resources of medicine, treating the boy’s sensation of a ‘breeze’ as a symptom permitted construction of an understanding of epileptic seizures. It demonstrates the action of the hermeneutical circle, as an experience missing from Galen’s prefiguration – the aura – permits refiguration of the understanding of seizures, with the purpose of guiding future treatment. Through symptomatisation, the aura becomes a candidate for medical explanation – and thence, intervention.

### Illness functions

If the above suggests some of the motivations clinicians have in translating illness experience into symptoms, it does not necessarily address what leads ill people to enter the clinical encounter in the first instance, to render their bodies and experiences subject to that act of interpretation.

At first, one might assume that their motivations are the same as those of the clinician: the clinician is an expert, and the ill person seeks their expertise to address (and hopefully ameliorate) their illness experience. However, the accounts of people living with some of the conditions perhaps least successfully managed with medical expertise – those with functional syndromes (or ‘medically unexplained symptoms’) suggest this is far from the primary motivation in seeking medical care. Qualitative research involving people with such conditions demonstrates that, by and large, they *do not* criticise clinicians for failing to diagnose or treat an underlying disease, or to palliate their symptoms (Peters et al. 1998). Instead, from the clinical counter they seek *acknowledgement* and *legitimation* of their suffering (Nettleton 2006; Salmon 2000). Patients whose experiences are not adequately recognised as symptoms worry about being a ‘fraud’, ‘time waster’, ‘hysteric’, ‘fake’ or ‘hypochondriac’ (Nettleton 2006); they seek naming of symptoms to legitimise them as forms of suffering, and to exculpate themselves from responsibility for the suffering (Barker 2017; Salmon 2000).

This function is strikingly demonstrated in the case of those who interpret their experiences symptomatically even in the absence of, or in conflict with, medical counsel – who undergo ‘self-medicalisation’. In a qualitative study conducted in France with people who had symptomatized their own experience, Sylvie Fainzang describes how – while they did use these symptomatic descriptions e.g. to guide self-medication – this did not appear to be the primary function of doing so. Instead, the symptoms were in each case linked to a perceived social or political harm (e.g. imposition of industrial infrastructure on the local environment, excessive work-related stress), and the symptom was required to legitimise the suffering caused by such harms:

*[S]elf-medicalization takes the form of a protest against the fact that people’s living and working environments have been taken over by commercial or industrial interests, by the pace and burden of work in the corporate world, or by societal choices. Self-medicalization thus equates to a condemnation of a particular social, economic, or political environment, and the medicalized sign becomes proof of the pathogenic nature of this environment.* (Fainzang 2013)

Through symptomatisation, they are permitted to give expression to their suffering at the hands of this ‘pathogenic environment’. That a hostile workplace, or the environmental degradation of one’s home for the ends of private industry, might be intolerable in their own terms is apparently inadequate evidence of wrongness within the societies supporting these arrangements. Viewing this suffering as symptom of a disease caused by such social or environmental conditions, however, places its legitimacy – and the demand to address it – beyond debate. Again the symptom is a commodity-actant, but with a very different exchange value, and demanding different ends, from that expected by the clinician. Thus symptoms to the ill person need not always serve the same function as they do to the clinician. This disparity in intended employment may be the source of breakdowns in the interpretive act of symptom construction, to which we now turn.

## Failures of symptom interpretation

With this framework in place, we can now begin to explore how the interpretation of experience as symptoms may go wrong. To do that, we can first introduce a – fairly straightforward – distinction, between symptom types and symptom tokens. Symptom types are the general sorts of things recognised as symptoms (e.g. ‘chest pain’, ‘nausea’, ‘anhedonia’), while symptom tokens are particular concrete instances of these types (e.g. the ‘niggling discomfort’ that is interpreted as chest pain in our opening example). With this distinction in play, we can recognise four distinct forms of interpretive failure in the process of symptom construction: the failure to recognise an experience as a particular symptom token; the failure to recognise a general form of experience as comprising a symptom type; the neglect of certain forms of human experience that are not adequately captured in trying to interpret them as ‘symptoms’; and feedback effects on people’s self-understanding that interpreting experience as symptoms may have.

### Failure of symptom-token recognition

The failure to interpret an ill person’s experience in terms of a particular symptom token is perhaps the one most familiar to clinicians and patients alike. Examples abound, and can take at least two forms: getting the ‘wrong’ interpretation (labelling someone’s ‘niggling discomfort’ as ‘indigestion’ rather than ‘chest pain’); or failing to acknowledge that an experience could be interpreted as a symptom at all (treating the ‘niggling discomfort’ as an expression of the ‘worried well’). Elyn Saks describes how her expression of her experience was, due to her history of psychotic illness, not recognised as describing the ‘worst headache of her life’ – terms that would make any clinician immediately concerned for the subarachnoid haemorrhage she had in fact suffered (Saks 2008). Havi Carel and Gita Györffy narrate the case of a 5-year old girl whose clinicians go on a wild goose chase for the causes of ‘double vision’, because they fail to interpret accurately her description of ‘blurred vision’ (Carel and Györffy 2014). Descriptions of ‘dizziness’ are notorious amongst clinicians for being difficult to interpret appropriately – and even clinicians most experienced in managing ‘dizziness’, fully aware of this difficulty, still systematically misread them (Sommerfeldt et al. 2021).

It is tempting here to assume that there is a ‘correct’ interpretation of certain experiences as tokens of certain symptoms. Saks’ clinicians failed her because they did not interpret her experience as representing ‘the worst headache ever’; the clinician who reads the ‘niggling discomfort’ as ‘indigestion’ fails because they do not recognise a report of ‘chest pain’ as indicative of cardiac disease. On this picture, symptomatic “complaints may be mapped directly onto sensations and pathological processes” (Good and Good 1981, p. 165). There is an underlying biological process, which reliably produces a certain experience; the way that experience is described by the ill person may be subject to certain ‘distortions’ of culture, social situation, or linguistic ability, but ultimately the clinician need simply unpick these distortions to reveal the underlying biological reality. This picture, however, is inadequate for both ontological and practical reasons.

Ontologically, it commits us to a strong, arguably eliminativist, claim about the nature of experience – that our experiences *just are* the phenomenal consequences of these biological processes; this commitment does not seem to respect our intuitions surrounding such experiences. If, for example, a person suffering a heart attack were to say they do not have any form of pain, would we say they were *mistaken* about their experience? It is for reasons such as this that, *inter alia*, the International Association for the Study of Pain define pain as “a personal experience” and explicitly state that “pain and nociception are different phenomena” (Raja et al. 2020).

Without even engaging in such metaphysical debates, however, there is a purely practical reason for not adopting this approach: even if first-personal experiences can in principle be reduced to biological processes, in clinical practice best evidence of them comes from phenomenal reports. The identification of symptom-tokens with certain biological processes paradoxically makes it *harder* for the clinician to predict what those processes might be. It is more useful to be able – when confronted by a person describing ‘the worst headache ever’ – to be able to evaluate how likely it is that the person has suffered a subarachnoid haemorrhage, i.e. to consider the symptom token an imperfect predictor of an underlying disease – than to say that either the person has suffered a subarachnoid haemorrhage, or else they are mistaken in reporting their experience/the clinician has failed to interpret their experience as the correct symptom-token.

This does, however, create some difficulty in deciding how the interpretation can be *mistaken*. If there is a gold reference standard – such as an underlying physiological process – then we can easily describe precisely wherein this failure lies, simply by comparing the interpretive performance against the gold standard. But we have just suggested there is no such gold standard. However, we can construct an alternative explanation of how we can fail to acknowledge symptom-tokens correctly in the clinical encounter, with reference to the functions symptoms are supposed to play in that encounter. In Elyn Saks’ case, her experiences were interpreted as ‘distress associated with psychotic illness’ rather than ‘worst headache ever’. Thus this symptom-token did not motivate her clinicians to pursue certain courses of action (e.g. obtaining a CT scan); it did not serve as a tool for communication (with radiologists or neurosurgeons); it could not adequately explain her experience (why would it differ so drastically from the previous manifestations of her schizophrenia?) The counterfactual story in which her experiences were interpreted as being of ‘the worst headache ever’ and led to investigation for and subsequent diagnosis of a subarachnoid haemorrhage is one in which the act of symptom-interpretation more successfully serves the clinical functions of that interpretive act.

### Failure of symptom-type recognition

Experiences may not just fail to be interpreted as belonging to a particular type of symptoms, however; aspects of illness experience may fail even to be recognised as potentially comprising a symptom type. By this I mean that certain forms of illness experience – though potentially relevant to serving the functions of symptoms described above – are not incorporated within the conceptual resources of medicine such that they can be adequately recognised as describing a distinctive way of being within a particular illness.

A potentially illustrative example is given by the phenomenon of ‘brain shivers’ or ‘brain zaps’ some people report on cessation of treatment with certain antidepressants. Since the early 1990s, reports can be found on regulatory agencies’ side-effect report records of brief ‘electrical shock’ or ‘flash’-like sensations experienced by some people when reducing or stopping treatment with antidepressants. With increasing access to the Internet, the terminology of ‘brain zaps’ became familiar amongst people using such medications and sharing their experiences – identifying a distinctive, intersubjectively-reproducible form of bodily experience. However, the recognition of such phenomena – indeed of antidepressant discontinuation syndromes in general – did not become common amongst clinicians until many years later. Patients experiencing these phenomena report clinicians ‘seeming bewildered’, or having ‘never heard of’ such experiences (Papp and Onton 2018).

In circumstances such as these, it appears that ill people are confronted by failure to recognise a symptom-type. The account of ‘brain zaps’ suggests that there is at least *prima facie* reason to believe that they may fruitfully be understood as theoretical entities of a biomedical model; and that they may successfully be managed by palliative symptomatic intervention (e.g. slower weaning of antidepressants); thus they are good candidates for being acknowledged as symptoms. But clinicians lacked the conceptual resources to parse them as such, resulting in breakdown in the interpretative activity of the clinical encounter.^7^

There are reasons to believe that certain archetypal characteristics of illness experience may result in their being particularly vulnerable to this problem. Two of these are highlighted by Havi Carel and Ian James Kidd, who note that much illness experience is characterised by *inarticulacy* – “the difficulty of adequately communicating, sharing, or ‘getting across’ certain aspects of the experience of illness” – and *ineffability* – “the sense that certain aspects of those experiences cannot be adequately communicated to others through propositional articulation at all because understanding is premised upon a person’s having had the requisite bodily experiences”(Kidd and Carel 2017). To these we can add that experiences, while being neither ineffable nor inarticulable, may nonetheless be *unspeakable* – inexpressible because the social penalties of doing so are considered too high; or ill persons might find clinicians *wilfully hermeneutically ignorant* of their experiences – while they can be successfully communicated in certain contexts, disproportionate epistemic privileges in defining the conceptual resources of the clinical encounter, and neglect of the testimony e.g. of certain oppressed groups, means that clinician audiences are ill-equipped to hear these accounts.

The narratives of people who experience seizures provide striking examples of the first three forms of vulnerability. As already explained, people with epilepsy – a tendency to recurrent epileptic seizures – will have recurrent, highly-stereotyped episodes of experiential or behavioural alteration. Yet despite this familiarity, it is a striking feature of communication with people with epilepsy that they frequently find these experiences inarticulable. They will say ‘this is hard to describe’, or ‘I don’t know how to say this’ (Schwabe et al. 2008); moreover, linguistic studies have demonstrated that their attempts to relate these experiences are marked by a very high degree of ‘formulation effort’ – hesitations, false starts, rephrasings and recapitulations, as they struggle to put into words what it is like for them to have a seizure (Pevy et al. 2021; Schwabe et al. 2007, 2008). But if experiences cannot adequately be communicated, it is difficult for the experience to be recognised as a potential symptom-type.

This is even more the case for ineffable experiences. Certain forms of seizures – e.g. so-called ‘ecstatic’ seizures – are commonly associated with experiences that those who have them not only struggle to describe, but feel that language would necessarily be inadequate to capture (Åsheim Hansen and Brodtkorb 2003; Greyson et al. 2015). They may draw from spiritual, religious, or erotic metaphor to articulate parts of this experience (Coles 2013), but ultimately find that the phenomenon is situated so far outside the normal realms of intersubjectively understood human experience that it cannot be shared, only felt. Clearly, if – as Carel and Kidd argue – it is a feature of many more forms of illness experience, ineffability poses a non-contingent barrier to identification of a potential symptom type.

Even when a person’s experience is in principle communicable and they have the means to articulate it, they may find themselves unable to share it in the clinical encounter for other reasons. This is the case in *unspeakable* experiences – those that, for reasons of taboo, anxiety about identity-shaping consequences, or other social reasons, people cannot bring themselves to express. The alien nature of epileptic experiences makes them perhaps particularly vulnerable to this problem – the authors of one paper describing an attempt to catalogue an exhaustive inventory of seizure-related symptoms note that, on inquiring about certain symptoms, participants often displayed a “strong emotional response” at being able to share an experience they otherwise felt unable to – from embarrassment, fear of being thought mad, or other reasons of stigma (Devinsky et al. 1991). In other forms of seizure, this unspeakable nature may not just be characteristic, but even causal; one suggested contributor to the pathogenesis of dissociative (nonepileptic) seizures (episodes of altered behaviour, awareness, or control that superficially resemble epileptic seizures but represent an unconscious reflex-like response to overwhelming sensory, cognitive, or affective stimuli rather than being the manifestation of excessive and hypersynchronous neuronal activity in the brain) is that the inability to communicate ‘unspeakable dilemmas’ results in their expression through bodily manifestations (Griffith et al. 1998).

A last concern about the potential for symptom types not to be recognised relates to the prejudices or prefigurations that shape what makes sense in the clinical encounter. As discussed earlier, what is intelligible in any given context depends in part upon the conceptual resources shared by the conversants in that context. We continually act to shape these resources; but differently-situated people are differently-able to do so effectively. People from certain situations, within certain contexts, may therefore find their understandings of certain phenomena excluded from other contexts, due to the disproportionate epistemic privilege other parties may enjoy in defining the conceptual resources of that context (Fricker 2009). This produces the possibility of what Gail Pohlhause Jr calls *wilful hermeneutical ignorance*:

*In the case where a marginally situated knower notices that dominantly held epistemic resources are not suitable for knowing her experienced world, dominantly situated people can dismiss both the possibility that there is anything to be known here and any epistemic resources that might have been developed to make sense of the experienced world of those marginally situated (*Pohlhaus 2012, p. *728)*.

It is not uncommonly alleged that clinicians and medical institutions enjoy just such a disproportionately inflated position in the clinical encounter (Ho 2011; Wardrope 2015). This may be amplified by the stigmatisation of particular presentations (such as seizures), or overlay on existing power dynamics (e.g. some conditions will disproportionately affect people from some marginalised communities). One (perhaps somewhat uncharitable to the clinicians) reading of the initial position with respect to ‘brain zaps’ is of wilful hermeneutical ignorance: that people taking antidepressants had created the concept and terminology to share with each other this particular experience associated with antidepressant discontinuation, but their epistemically marginalised position within psychiatric discourse imposed barriers to incorporation of these resources into the clinical context. Such a reading would represent a case of failure of symptom-type construction due to wilful hermeneutical ignorance.

### Loss of the lived body

The first two types of hermeneutical failures are ones that most clinicians are familiar with and readily acknowledge: that sometimes they ‘read the symptoms’ incorrectly, or fail even to appreciate that a patient is describing something that could be usefully treated as a symptom. The next putative hermeneutical failure, however, challenges the very rationale of the interpretative activity that renders illness experiences as symptoms.

Such accusations generally proceed from the starting point that symptoms are attempts to render first-personal experiences accessible and intelligible in third-personal terms, and thence incorporate them into objective models of disease phenomena. In Sartre’s terminology introduced earlier, symptoms are features of the ‘body-for-the-Other’. Others working in the phenomenological tradition identify this with what Edmund Husserl calls ‘*Körper’* and Maurice Merleau-Ponty ‘*le corps objectif*’; in each case, these are contrasted with the first-person, phenomenal ‘body-for-me’/’*Leib*’/’*corps propre’* Carel 2016; Husserl 1989, p. 46; Merleau-Ponty 2002; Sartre 1978). The concern is then raised that the emphasis on the translation of experience into symptoms in the clinical encounter prioritises this objective body over the subjective or lived body of illness, and in so doing obscures vital aspects of the ill person’s experience. By focusing on the construction of symptoms, it is alleged, medicine creates a situation whereby clinician and patient are two parties divided by a common language, trying to describe two different things – the former the disease process within the objective body, the latter the illness experience within the lived body – in the same terms (Carel 2016, p. 47).

This accusation may seem odd at first blush – if the interpretive act of symptom construction is a perspectival one, designed to serve certain functions as described above, we should hardly expect it to analyse the experience of illness completely and without remainder; its concern is simply those aspects of the experience that are most relevant to serving the functions of symptoms. And as far as the clinical functions are concerned, it is the objective body that is most relevant. To serve as entities within actual or theoretical biomedical models is necessarily to make symptoms part of the objective body; to serve as tools for communication, or evidence-based guides for palliative intervention, then the descriptions must at least be interpersonally accessible and reliably intelligible, which some of the specific features of illness experience discussed in the previous section would make them unsuited for.

This critique, then, is best read as an opposition to the *exclusive* importance of interpreting illness experience in terms of symptoms. Even with this reading, though, there is a case to be made for the primacy of symptoms as interpretation of illness experience, *within the clinical encounter*; for clinicians are experts in the diagnosis and treatment *of disease*. Patients, when presenting their problems in a clinical context, typically readily hand the role of framing the discussion to clinicians as soon as given opportunity; by consulting medical expertise they are *seeking* a medicalized explanation (Beckman and Frankel 1984; Heritage 2009). That there are many aspects of illness for which they lack the relevant expertise to manage is not a case for trying, as in Goethe’s turn of phrase, to turn the world into one giant hospital; rather, it is to acknowledge that when we consider what illness means in other aspects of our personal and social lives, we need to look to resources other than those that can be offered by medicine. To let the centripetal force of medicalised descriptions dominate our understanding of illness experience neglects the ‘surplus of meaning’ within it, and misunderstands the perspectival nature of medical interpretation – it mistakes the map for the territory (Wardrope 2017).

### Shaping experience of the intersubjective body

The last purported failure of the interpretation of symptoms is less apparent in the individual clinical encounter, but more a downstream consequence of its taking place – with cumulative effects across time and across people. To illustrate this effect, we can return to the case of Sartre’s gastralgia discussed earlier. The story as we left it – in which the unnamed aching in the ‘body-for-me’ was transformed into the dyspepsia of the ‘body-for-the-Other’, causally linked to the stomach ulcer within the clinician’s model of the objective body – was incomplete. For Sartre describes how the transformation of illness experience into symptom not only constructs the objective body that is present for others; there is a feedback effect whereby the original illness experience comes to be reinterpreted in light of the objectified description of the pain as dyspepsia:

*Thus the injured stomach is present through the gastralgia as the very matter out of which this gastralgia is made. The stomach is there; it is present to intuition and I apprehend it with its characteristics through the suffered pain. I grasp it as* that which is gnawed at. (Sartre 1978, p. 357)

Thus, through the act of the clinician’s interpretation, the ill person’s relation to their own experience is changed; they experience their body not only – perhaps even not primarily – as the ‘body-for-me’, but as the ‘body-seen-by-the-Other’. They understand their experiences in the ways that others have made them intelligible. But, as already covered, this perspective was never intended to incorporate everything important about the experience of illness. This line of critique, then, worries that if the body-seen-by-the-Other comes to dominate the body-for-me, it might obscure certain aspects of ill people’s experience as their self-interpretation becomes overwhelmed by medicine’s.

To put this worry in hermeneutical terms, the interpretation of illness experience as symptoms may feedback (via the hermeneutical circle) into our prior expectations, to the point that the symptomatic representation dominates our self-understanding of future experiences. On the predictive coding model, the symptomatic description would dominate our priors for our own bodily experience; thenceforth, the body as perceived would be as much an artefact of what it is expected to be, as what sensory input tells us of it (Edwards et al. 2012; Van den Bergh et al. 2017).

Again, turning to the histories of people who experience seizures, we can see potential examples of this in effect – indeed, in ways which even prevented or delayed recognition of experiences that could be usefully interpreted as symptoms. Seizures are typically described as *paroxysmal* – occurring ‘out of the blue’ – and *unpredictable* – while certain factors (e.g. sleep deprivation, medication changes) may make them more likely, one cannot reliably say when any given seizure will occur. This model has resulted in seizure experiences being similarly described in such terms – the sudden onset of altered experience, behaviour, or awareness. People with seizures – who come to understand their condition through this model – will habitually describe their experiences in such terms. In a series of studies using explicitly phenomenological interview techniques to explore peri-ictal experience, however, Claire Petitmengin and colleagues demonstrate that many people – given the right environment – can articulate a more diffuse prodrome – often of fatigue, or being ‘ill-at-ease’ – for as long as 24 h before a clinical or electrographic seizure occurs.(Petitmengin 2006; Petitmengin et al. 2006, 2007) Obtaining these reports, however, often proved challenging – precisely because their participants implicitly understood their experiences in terms of seizures as paroxysmal events:

*the whole of medical discourse on epilepsy is underpinned by the belief that seizures are sudden, that they cannot be anticipated or prevented by the patient. We have observed that this belief considerably hampered the awareness and the description by the patient of the early symptoms that could enable him to anticipate and manage his seizures*. *(*Petitmengin 2006, p. *235)*

Petitmengin’s hypothesis here is that medicine’s describing seizures in a certain way conditions those who experience seizures to think of them in such terms; this obscures aspects of that experience that fit less neatly into that model. A similar process is described in still more vivid detail in some accounts of living with mental illness. Katie Aubrecht writes:

*Under the watchful gaze of a physician, I was taught to read experiences, red cheeks, heavy hearts, and knots, as symptoms of mental illness and as tests of my character. I was constantly quizzed about how well I knew the experiences I had were actually true experiences. I couldn’t be sure what I felt, liked, or wanted anymore. I did, however, become ever more familiar with what doctors felt, liked, and wanted, and that those would be the right things to feel, like, and want*. (Fabris and Aubrecht 2014, p. 190)

In these cases, the entirety of the ill people’s experiences are subsumed by what medicine makes of them and can meaningfully say about them. The act of interpretation has the side-effect of producing “the successful and unfortunate transformation of a mystery into a graspable problem”(Eriksen and Risør 2014), whereby what is intelligible (within a particular context, for particular ends) implicitly becomes all that there is to understand. The ill person in these examples experiences what Gaile Pohlhaus Jr describes as a “truncated subjectivity” (Dohmen 2016; Pohlhaus 2014), whereby

*she is treated as if her own lived experience from which she draws in order to add to the communal knowledge pool is simply a mirror (or perhaps a shadow) [of dominant explanations of experience] … but certainly not capable of contributing to our understanding of the world beyond.* (Pohlhaus 2014, p. 106)

The case for this occurring in medicine is perhaps overstated in some of the literature on medicalisation; the totalising picture of the Foucauldian ‘medical gaze’ neglects the centrifugal forces on display within ill people’s utilisation or bargaining with medicine to develop their own self-understandings (Chin-Yee et al. 2020; Lock 2001; Wardrope 2015); but nonetheless the cases above highlight it as a potential adverse effect of the hermeneutics of the clinical encounter.

## Remedying failures in the interpretation of symptoms

So far, I have argued that the interpretive activity of constructing symptoms from illness experience is a crucial component of the clinical encounter; that the symptoms thus constructed serve a range of clinical functions, and these may not necessarily align with the functions the ill person assumes they will have; and that reading symptoms off experiences is far from a straightforward activity, but rather a complex, messy and iterative act that can go wrong in a variety of fashions. To conclude, I will suggest some potential measures that might be taken from the clinician’s side to ameliorate some of these challenges.

These measures comprise a mixture of tools and attitudes. Tools are things that clinicians may pick up and use to achieve certain ends in their practice. Like tools in any other context, they can be misused if misappropriated to the wrong contexts or wielded insensitively. Attitudes, meanwhile, are more ways to be – attributes and perspectives that can be cultivated through reflection and practice (if wishing to confine ourselves to a particular model of moral clinical practice, one could consider them ‘virtues’).

We are seeking to give an account of how human experiences might successfully be interpreted as symptoms. But some of the purported failures of this interpretive act described above - ignorance of the lived body, or overwhelming it through shaping the intersubjective body – make symptom-discourse itself (or its disproportionate epistemic authority) the problem. In these critiques, it is the assumption that the objective body of medical discourse describes reality – and thus the totality of experience – that leads to obscuration of aspects of experience not adequately captured therein. Thus in attempting to redress this imbalance, we might start with tools that begin from the suspension of this assumption.

It is, of course, the suspension of this default naturalistic representation of experience – the *epoché*, in Husserl’s terminology – that forms the departure point of phenomenological inquiry (Gallagher and Zahavi 2020). Various authors have suggested how incorporating such phenomenological investigation into the clinical encounter may help address these interpretive shortcomings. As referenced above, Claire Petitmengin describes how experiences rendered obscure by the dominant interpretations of illness can be brought forward by the technique she calls ‘microphenomenology’, a set of interviewing tools designed to keep interviewees in the moment of recalling the peripheral dimensions of a singular experience (Petitmengin 2006; Petitmengin et al. 2019). From the patient perspective (though she also advocates its use by clinicians), Havi Carel proposes the employment of a ‘phenomenological patient toolkit’ that can serve to “enable the expression of unique personal experiences rather than pushing patients to adapt their experiences to medical or cultural expectations” (Carel 2016, p. 202). She describes how use of the toolkit in a workshop setting explicitly helped to address the concerns raised about the subsumption of the lived body in the intersubjective, that it “would enable patients to take responsibility for their understanding of illness by enhancing their self-knowledge”(Carel 2016, p. 203). Microphenomenological interviewing, meanwhile, has previously been used to articulate aspects of seizure experience obscured by medicine’s implicit ontology of seizures. This has not only enhanced patients’ self-understanding, but also served to refine our models of seizure initiation – in other words, overcome barriers to symptom-type recognition as well (Petitmengin et al. 2006, 2007).

While this first tool suspends the ‘natural attitude’ in its exploration of experience, a complementary approach seizes on the intersubjective nature of experiences that could fruitfully be rendered ‘symptom’ to use existing symptom-types to help people articulate their own experience. In failures of symptom-token recognition, we saw how barriers to interpretation (whether simply of misfortune – reading ‘chest pain’ as ‘indigestion’ – or conditioned by structural injustice – paying less credence to the testimonial style of those with serious mental illnesses, such as in Elyn Saks’ case) could result in missing or misidentifying the most relevant symptoms to describe an ill person’s experience. In failures of symptom-type recognition, we saw that difficulties finding the appropriate language to describe symptoms – inarticulacy – or in having the social licence to express them – unspeakability – could inhibit kinds of experience being parsed as potentially representing symptoms in the first place. These can potentially be ameliorated by drawing from other people’s similar experiences to provide patients with the language to articulate their experiences – presenting them with a menu of possible interpretations, seeing which fits best, and jointly constructing the symptomatic account.

Clinicians to an extent often learn to do this intuitively: presented with the ‘dizzy’ patient, for instance, they will ask: do you mean dizzy like you stood up too fast, or are going to collapse (looking for a ‘presyncopal’ symptomatisation); or like you’re drunk, or seasick, or the world is spinning around you (a ‘vertiginous’ presentation); or like the world is warped, unreal, or you’re detached from it (‘dissociative’). They thereby present the patient with the means of refining their own account of their experience in a way that might best direct the process of symptom construction in order effectively to serve the ends of the clinical encounter.

However, the intuitive and ad-hoc implementation of this approach will still be vulnerable to clinician oversights and biases – Elyn Saks’ clinicians may have presented her with a range of possible interpretations, but failed to include any that would sufficiently associate her presentation with the possibility of a subarachnoid haemorrhage. One means of potentially addressing this is to use more systematic means of presenting possible symptoms – for example, through the use of checklists or questionnaires. Checklists are increasingly widely used throughout medicine and can successfully address certain oversights and biases in clinical reasoning and decisionmaking (Pronovost et al. 2006; Wolff et al. 2004). In this context, review-of-symptom checklists or questionnaires – tools presenting a range of possible interpretations of people’s experiences – can be used to attempt to capture a systematic profile of potential experiences. This may help mitigate against biases that could lead clinicians not to inquire about certain symptoms, or to dismiss them if reported. Such questionnaires tend to produce a greater symptom count than with open questioning alone, suggesting they may help provide the means for people to articulate their experience (Devinsky et al. 1991). Furthermore, they signal to the ill person that the clinical context might be one where it is safe to attempt to express certain experiences, that they are not taboo – and so render the unspeakable, speakable (Devinsky et al. 1991). Systematic inquiry about such symptoms can improve rates of accurate diagnosis over unguided clinical assessment, suggesting that they can play a role in serving the intended functions of symptoms (Reuber et al. 2016; Wardrope et al. 2019).

This is not to say that such tools will necessarily allow ill people to communicate all that they wish to of their experiences. Qualitative studies with people invited to complete symptom questionnaires highlight the tension between centrifugal and centripetal forces in symptom-interpretation – responses ranging from endorsement to rejection, through confusion and reinterpretation (Malpass et al. 2021). They may respond to perceived inadequacies of these tools by reformulating their items, recontextualising them, or explicitly challenging the conceptualisation of their experience presented therein (Galasiński 2008).

This is hardly surprising, given the different kinds of failures possible in the interpretation of symptoms highlighted above. I have suggested that questionnaires may assist in symptom-token identification – failures in which are failures by the standards internal to symptom-discourse. I do not pretend that tools such as these are able to solve the complex, messy, and imperfect nature of this activity. As already described, they can also, if used carelessly,^8^ make things worse rather than better. A last potential measure to confront difficulties in the interpretation of symptoms, then, is to cultivate an attitude towards the construction of clinical knowledge that shows an awareness of these limitations, and seeks out alternative resources for their remedy.

Multiple authors have summarised this as an attitude of *epistemic* or *intellectual humility* (Buchman et al. 2017; Ho 2011; Lakeman 2010; Wardrope 2015). While there is extensive debate on how best to analyse humility in general, and epistemic humility in particular (Alfano et al. 2020), a useful sketch can be found in the definitions applied in the psychological literature, in which it is modelled as a “willingness to recognise the limits of one’s knowledge and appreciate others’ intellectual strengths” (Porter and Schumann 2018). This definition contains both a reflexive, evaluative part – the ability to assess one’s own epistemic limitations accurately – and a dispositional, relational part – a tendency to seek sources outside the self in seeking to overcome those limitations (Ho 2011; Wardrope 2015). The epistemically humble clinician in this sense will acknowledge: that the process of constructing illness experience as symptoms, while inherent to the success of the clinical encounter, is difficult and prone to error; that the model of the patient’s disease thus constructed tells only part of the story of their illness, and for many aspects of their life – including decisions to be made about ongoing management – this may not be the most relevant part; and that by exploring other aspects of the ill person’s experience – in which the clinician does not necessarily have relevant expertise, and is better placed to learn from than to cast judgment upon – important lacunae in the medical picture of that experience may be addressed.

Epistemic humility, understood in this way, can improve our epistemic capacities. The epistemically humble are more open to opposing views (Porter and Schumann 2018), and tend to evaluate strong or weak arguments for positions more accurately (Leary et al. 2017). It can be cultivated – in the individual, for example by learning to adopt a ‘growth’ mindset towards our intellectual capacities (Leary et al. 2017); and collectively, by reducing threats to evaluators’ perceived epistemic competence (Tjosvold et al. 1980), or the stakes placed in their being perceived as ‘right’ or in an epistemically superior position (Porter and Schumann 2018). This suggests that epistemic performance in clinical practice could be improved by giving clinicians the space to be wrong. Allowing clinicians to acknowledge the limitations of their knowledge and the uncertainties inherent in clinical practice may help to cultivate epistemic humility. This requires tempering expectations from patients – who consistently rate clinicians as less competent and interactions less satisfying when they express higher uncertainty (Johnson et al. 1988; Ogden et al. 2002) – and resisting the expropriation of medical understandings of illness experience outside the contexts for which they were designed (thus, e.g., not assuming that a medical description is the most relevant for determing one’s legal liability, or legibility for social security support or environmental modifications to enable ability (Ho 2011; Szmukler 2014; Wardrope 2015)).

## Conclusions

The clinical encounter starts with an experience of illness. But that experience is not transparent or readily accessible even from the first-person perspective, let alone the third-person. The function of the clinical encounter is to support people in navigating their illness experiences, through employment of clinicians’ specific expertise – interpreting and managing that experience in terms of disease of the objective body. To engage in this pursuit requires a first, basic interpretive activity – the translation of experience into symptom. In this paper, I hope to have offered a justification of considering this interpretive activity as a site worthy of philosophical and clinical attention, to understand better: how such interpretations are achieved; what can go wrong in the process; and how such failures might be ameliorated.

## Footnotes

1 I am grateful to Sophie Williams for making this point.

2 As addressed further below, I focus on the clinical functions here, though undoubtedly presenting experiences as symptoms can have a broader social role in other contexts.

3 In fact, as we shall see later, this story is incomplete, omitting the feedback effects of this process that create the intersubjective order.

4 While it might be argued that some interventions (particularly psychological therapies e.g. acceptance and commitment therapies, mindfulness-based practices) need not have a clear symptomatic target to be employed, there is nonetheless a sense in which these require symptomatisation: in order to recommend their application, one need at least say that: (a) there is a certain kind of experience being had; and (b) that experience is the kind that might fruitfully be modified by employing such therapies. In this recognition, the experience is effectively treated as symptom (even if not explicitly categorised as such).

5 It is important to note that, while I have labelled this approach ‘palliative’, the discipline of ‘palliative care’ is entirely distinct (though of course it may employ this approach, as any other branch of medicine) (Cellarius and Upshur 2014; Genuis 2017).

6 The role of symptoms as commodity-actants is if anything even more clearly demonstrated in the many cases of people admitted to Stroke Units who are subsequently found *not* to have suffered strokes. Frequently clinicians – whether they be paramedics, emergency physicians, or GPs – will be confronted by people describing sensations or displaying bodily changes that are difficult to parse clearly, but that *could* be interpreted as symptoms suggestive of stroke (‘slumping to the right’ could represent ‘right-sided weakness’; ‘mumbling and muttering’ *might* be ‘expressive dysphasia’). Working in time-pressured environments, with limited means of gathering further information, they nonetheless have to elect a definitive course of further action, and communicate successfully in order to ensure it is enacted. This is more likely to be successful when the patient’s presentation is identified with a relevant symptom with appropriate commodity value; the Stroke Team are more likely to accept the ‘expressive dysphasia’ than the person who is ‘mumbling and muttering’.

7 Post-chemotherapy cognitive impairment (‘chemo fog’ or ‘chemobrain’) may represent another instance of a similar phenomenon, an example for which I am grateful to Sophie Williams for highlighting (Hede 2008).

8 Or out of context – a detailed exploration of the phenomenology of stroke might delay the pressing need for emergent intervention!

## Statements and declarations

This work was undertaken during an Academic Clinical Fellowship funded by the National Institute for Health Research (NIHR) (AW). The views expressed are those of the author(s) and not necessarily those of the NIHR or the Department of Health and Social Care.
